# An Analysis of the Mechanism About CO_2_ Enrichment Promoting Carbohydrate Metabolism in Cucumber (*Cucumis sativus* L.) Leaves

**DOI:** 10.3390/ijms252011309

**Published:** 2024-10-21

**Authors:** Yanling Li, Hongxia Song, Xuan Li, Leiping Hou, Meilan Li

**Affiliations:** 1 College of Horticulture, Shanxi Agricultural University, Taigu 030801, China; liyanling0355@163.com (Y.L.); 13834836584@163.com (H.S.); sxndhlp@126.com (L.H.); 2 School of Life Science, Shanxi Normal University, Taiyuan 030031, China; lx20130505130@163.com

**Keywords:** cucumber, CO_2_ enrichment, transcriptome, metabolome, glucose

## Abstract

Elevated CO_2_ can affect the synthesis and distribution of photosynthetic assimilates. However, the carbohydrate metabolism molecular mechanism of cucumber leaves in response to CO_2_ enrichment is unclear. Therefore, it is of great significance to investigate the key functional regulatory genes in cucumber. In this study, the growth of cucumber leaves under different CO_2_ conditions was compared. The results showed that under CO_2_ enrichment, leaf area increased, the number of mesophyll cells increased, stomata enlarged, and more starch grains accumulated in the chloroplasts. Compared with the control, the starch and soluble sugar content of leaves were maximally increased by 194.1% and 55.94%, respectively; the activities of fructose-1,6-bisphosphatase (FBPase), ADPG pyrophosphorylase (AGPase), starch synthase (SSS), sucrose phosphate synthase (SPS), sucrose synthase (SS) and invertase (Inv) were maximally increased by 36.91%, 66.13%, 33.18%, 21.7%, 54.11%, and 46.01%, respectively. Through transcriptome analysis, a total of 1,582 differential expressed genes (DEGs) were identified, in which the starch and sucrose metabolism pathway was significantly enriched, and 23 genes of carbon metabolism were screened. Through metabolome analysis, a total of 22 differential accumulation metabolites (DAMs) were identified. Moreover, D-glucose and D(+)-glucose were significantly accumulated, showing upregulation 2.4-fold and 2.6-fold, respectively. Through combined analysis of transcriptome and metabolome, it was revealed that seven genes were highly related to D-glucose, and *Csa6G153460* (AGPase), *Csa5G612840* (β-glucosidase), and *Csa4G420150* (4-α-glucanotransferase) were significantly correlated to the carbohydrate regulatory network. Furthermore, the mechanism of CO_2_ enrichment that promotes carbohydrate metabolism in leaves at the molecular level was revealed. This mechanism advances the development of the cell wall and leaf morphology by activating the expression of key genes and improving enzyme activity.

## 1. Introduction

CO_2_ is a substrate for photosynthesis, and its concentration directly affects the photosynthetic efficiency of plants, which indirectly affects plant growth and development by altering a series of physiological processes such as carbon and nitrogen metabolism and cell cycle changes, thereby affecting crop yield and quality [1]. During greenhouse production in winter, cucumber consumes a significant amount of CO_2_ during photosynthesis, resulting in a decrease in the CO_2_ content of the greenhouse. Additionally, the temperature is low in winter. Thus, adequate ventilation cannot be provided timely [2]. Therefore, it is necessary to investigate the effects of artificially increasing CO_2_ in greenhouse conditions to respond to physiological metabolism in leaf cells.

Carbon metabolism of CO_2_ is determined by a combination of photosynthesis, respiration, and carbohydrate storage and remobilization capacity [3]. Elevated CO_2_ increases carbon sources, leading to an accelerated rate of carbon assimilation in photosynthesis and providing more carbohydrates for plant metabolism [4]. The enhancement of photosynthesis under elevated CO_2_ can cause plants to produce more sugar, including glucose, fructose, and raffinose [3,5]. Under carbon-enriched conditions, the tomato showed a significant increase in net photosynthetic rate, as well as wider and thicker leaves, significantly improved leaf area, and significant increases in starch, soluble sugar, and sucrose contents in leaves. The soluble sugar of tomato fruit rose sharply during the ripening process, and the accumulated biomass of tomato plants increased [6,7,8]. Similarly, during the harvesting and post-ripening stages of sweet melon fruits, CO_2_ enrichment can promote an increase in soluble sugar, sucrose, and fructose content [9]. Moreover, some primary metabolites of the stem in *Arabidopsis* increased under elevated CO_2_, such as the levels of starch and triose phosphate, especially at the end of the light period [5]. These extra sugars could be used for the development of new sink organs, such as leaves, stems, tillers, and seeds, and the eventual growth and development of the plant is a result of the response of these new organs to elevated CO_2_ [1]. In general, the magnitude of the plant’s photosynthetic response to elevated CO_2_ varies with the stage of plant development and the prevailing environmental conditions [10]. The production and consumption of starch and sucrose, two photosynthetic products, is a dynamic process that also varies according to environmental conditions and plant metabolic needs. Therefore, elevated CO_2_ can affect the synthesis and distribution of photosynthetic assimilates.

Carbohydrate metabolism in plants involves the synergy of a series of enzymes. Fructose-1,6-bisphosphatase (FBPase) regulates the synthesis of starch and sucrose [11]. ADPG pyrophosphorylase (AGPase) and starch synthase (SSS) play a large role in controlling starch synthesis [12]. Sucrose phosphate synthase (SPS), sucrose synthase (SS), and invertase (Inv) are key enzymes of sucrose metabolism that regulate the biosynthesis and transportation of sucrose in most terrestrial plants [13,14,15]. Under elevated CO_2_ conditions, the starch accumulation and activities of SPS and SS increased during photosynthesis in rice, especially for fully mature young leaves [16]. The related specific genes of SS (*Rsus1*) and SPS (*Sps1*) in rice showed high expression [17]. The expression of genes participating in ribulose-1,5-bisphosphate (RuBP) regeneration and coding fructose-1,6-diphosphatase (FBPase), transketoase, and aldolase were upregulated under elevated CO_2_ [18]. Under elevated CO_2_, the expression of three carbohydrate metabolism genes in wolfberry fruit *LBGAE* (UDP-glucuronide-4-epimerase), *LBGALA* (α-galactosidase), and *LBM* (malate synthase) was significantly increased, regulating four carbohydrate metabolisms and lipid metabolism pathways, affecting the activities of sucrose-related enzymes [19]. In addition, in sugar cane, the genes of NADP-malate dehydrogenase related to photosynthesis photoreaction and pyruvate orthophosphate dikinase (PPDK) were adjusted by 117% and 174%, respectively, 14 days after the leaves emerged, and then, the genes relating to sucrose metabolism were adjusted, so the content of sucrose has been increased [20].

Cucumber is an important vegetable for facility cultivation, which adopts intensive production and has achieved an annual supply. At present, there is some existing research on CO_2_ enrichment promoting the growth and development of cucumber [21,22,23]. Most of the studies focused on the physiology and photosynthesis of cucumber seedling growth under CO_2_-enrichment conditions. However, for cucumbers produced in greenhouse facilities, the molecular mechanisms of carbohydrate metabolism in leaves in response to CO_2_ enrichment have not been examined. Therefore, this study investigates the growth of cucumber leaves, the carbohydrate content, and the activity of carbohydrate metabolism-related enzymes under CO_2_ enrichment. Moreover, we conduct transcriptome and metabolome analysis to correlate DEGs with DAMs and screen the key candidate genes of cucumber leaves in response to CO_2_ enrichment. The aim of this study is to reveal the mechanism of CO_2_ enrichment that promotes the growth of cucumber leaves at the molecular level and provide a certain theoretical basis for cucumber cultivation and breeding.

## 2. Results

### 2.1. Effect of CO_2_ Enrichment on the Growth and Development of Leaves

**Figure 1:**
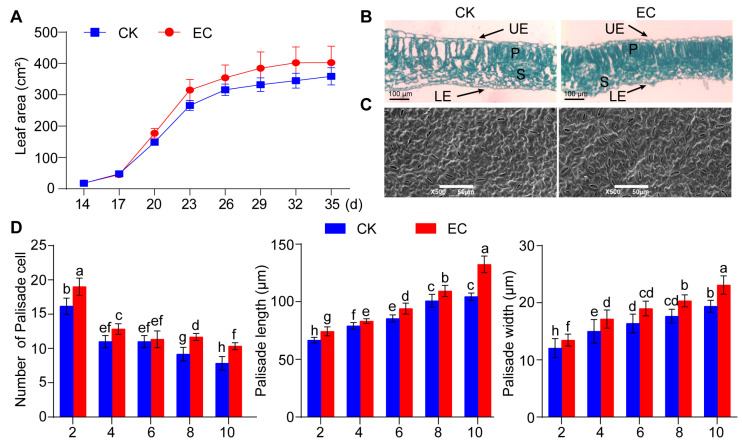
CO_2_ enrichment promotes the growth and development of leaves in cucumber. (**A**) Leaf area statistics of leaves under CO_2_ enrichment within 35 d. CK represents the natural condition, while EC represents CO_2_ enrichment treatment. (**B**) Microscopic observation of the mesophyll tissue structure of leaves under CO_2_ enrichment for 30 d. UE is the upper epidermis, LE is the lower epidermis, P is the palisade tissue, and S is the sponge tissue. The arrow indicates the represented position. (**C**) Observation of the epidermal structure in cucumber leaves enriched with CO_2_ for 30 d using scanning electron microscopy (×500). (**D**) Determination of the number, length, and width of palisade cells in cucumber leaves enriched with CO_2_ for 30 d. Significant differences indicated by different letters were calculated using Duncan’s new multiple range test (*p* < 0.05).

By dynamically analyzing the growth of leaves in cucumber cultivar under CO_2_ enrichment, it was found that the leaf area in the CO_2_-enriched zone was higher than that of the control (Figure 1A). On the 29th day of CO_2_ enrichment, the leaf area in the CO_2_-enriched zone showed a significant difference compared with the control, with a relative increase of 19.32%. When CO_2_ enrichment continued for 35 d, the leaf area in the CO_2_-enriched zone reached a maximum of 402 cm^2^. Upon observing the cross-sectional structure of leaves in cucumber (Figure 1B), it was found that the arrangement of mesophyll cells was loose in the control zone, while the arrangement of mesophyll cells was tighter and neater in the CO_2_-enriched zone, with an increase in volume and surface area. In particular, the number of palisade cells significantly increased. At the same time, under scanning electron microscopy, it was found that the stomata enlarged the leaves’ epidermal cells in the CO_2_-enriched zone (Figure 1C). This result indicated that CO_2_ enrichment can enhance photosynthesis by regulating stomatal opening, thereby promoting tissue development in cucumber leaves.

Subsequently, the characteristics of leaf mesophyll cells in cucumber were measured, and it was found that the number of palisade cells in leaves at the 2nd, 4th, 8th, and 10th nodes increased significantly compared with the control, by 14.2%, 16.4%, 30%, and 25% respectively (Figure 1D). Moreover, the number of palisade cells increased more obviously with increasing leaf age. At the same time, there was a significant difference in the size of leaf palisade cells at the 2nd, 4th, 6th, 8th, and 10th nodes in the CO_2_-enriched zone compared with the control. The length increased by 11.44%, 5.16%, 10.05%, 8.42%, and 26.87%, respectively, while the width increased by 16.9%, 14.33%, 16%, 15.3%, and 19.07%, respectively. These results showed that CO_2_ enrichment significantly promoted the growth of leaves in cucumbers.

### 2.2. Effect of CO_2_ Enrichment on Carbohydrate Accumulation and Enzyme Activity of Leaves

**Figure 2:**
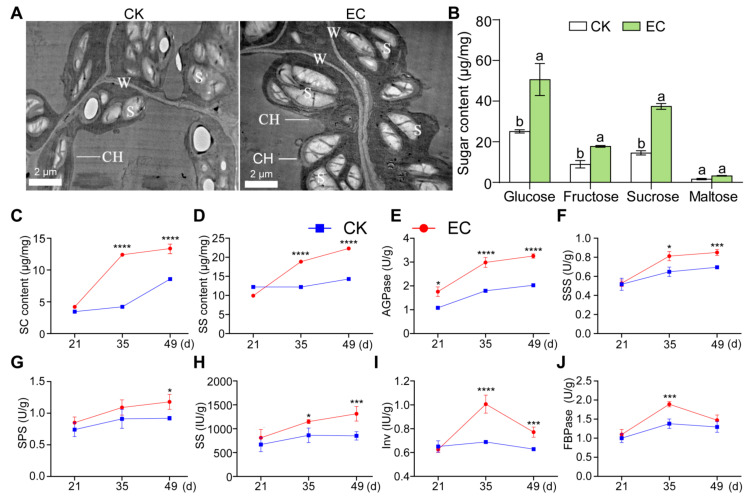
Accumulation of starch and soluble sugar in cucumber leaves under CO_2_ enrichment. (**A**) Observation of starch accumulation in chloroplasts on the 30th day of CO_2_ enrichment by transmission electron microscopy (×10 k); W is Cell wall; CH is Chloroplast; S is Starch grain. (**B**) Determination of sugar (sucrose, glucose, fructose, and maltose) content on the 35th day of enriched CO_2_. Duncan’s new multiple range test was used to calculate significant differences; a and b represent the level of significant difference (*p* < 0.05). (**C**,**D**) The content of starch (**C**) and soluble sugar (**D**) during continuous CO_2_ enrichment for 49 d. (**E**–**J**) The activities of AGPase (**E**), starch synthase (**F**), sucrose phosphate synthase (**G**), sucrose synthase (**H**), invertase (**I**), and fructose-1,6-diphosphatase (**J**) in leaves during continuous CO_2_ enrichment for 49 d. Asterisks indicate significant differences using the two-tailed Student’s *t*-test (* *p*  <  0.05, *** *p*  <  0.001, and **** *p*  <  0.0001).

In order to analyze the mechanism of CO_2_ enrichment to promote the growth of cucumber leaves, the chloroplasts of cucumber leaves enriched with CO_2_ for 30 d were observed using transmission electron microscopy. The chloroplasts of cucumber leaves under CO_2_ enrichment were observed to be enlarged and distributed closely to the cell wall, especially, while the number of starch grains in the chloroplasts increased significantly and the volume increased compared with that of the control (Figure 2A). Subsequently, the starch content of leaves was measured, and it was found that starch accumulated under CO_2_ enrichment more than the control (Figure 2C). The content of starch increased by 21.68%, 194.1%, and 55.94%, respectively, on the 21st, 35th, and 49th days under CO_2_ enrichment. The changes in starch content were especially obvious under CO_2_ enrichment on the 35th day. The results showed that the starch accumulated the most in carbohydrates, and the effect of the middle stage of the CO_2_ treatment was significantly better than that of the first and later stages.

At the same time, the accumulation of soluble sugar in cucumber leaves gradually increased (Figure 2D). When CO_2_ was enriched for 35 d and 49 d, the soluble sugar content of the leaves increased by 54.63% and 55.94%, respectively, compared with the control. By determining other sugar content in leaves (Figure 2B), it was found that there was no significant difference in maltose content, and the contents of glucose and sucrose were significantly higher than that of fructose and maltose, indicating that CO_2_ enrichment significantly promoted the synthesis of sucrose, glucose, and fructose in cucumber leaves.

The carbohydrate metabolism of leaves in cucumber is a complex process, which is regulated by a series of enzymes. After CO_2_ enrichment, the activities of four key enzymes involved in carbohydrate metabolism, AGPase, SSS, SPS, and SS, in cucumber leaves gradually increased with the extension of CO_2_-enrichment time (Figure 2E–H), and the activities of two other enzymes, FBPase and Inv, showed a decreasing trend after CO_2_ enrichment for 35 d (Figure 2I,J). Compared with the control, the activity of FBPase-regulating starch and sucrose synthesis was the highest on the 35th day of CO_2_ enrichment, which increased by 36.91%; the activities of AGPase and SSS relating to starch synthesis reached the highest on the 49th day of CO_2_ enrichment, which increased by 66.13% and 33.18%, respectively; and the activities of SPS, SS, and Inv relating to sucrose metabolism maximally increased by 21.7%, 54.11%, and 46.01%, respectively. The results showed that AGPase activity increased the most, which may be the cause of starch accumulation.

### 2.3. Transcriptome Analysis of Leaves under Different CO_2_ Conditions

**Figure 3:**
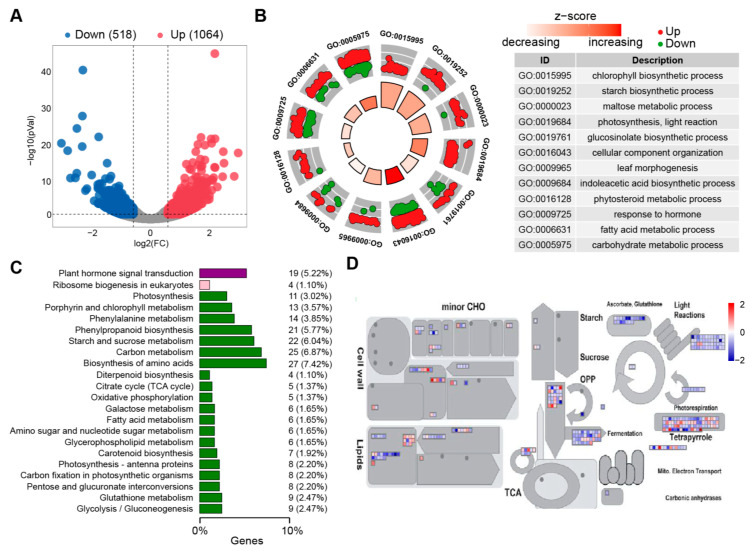
Enriched analysis of DEGs in cucumber leaves under different CO_2_ conditions. (**A**) Volcanic diagram of DEGs. Gray dots show non-significant. (**B**) GO functional enrichment circular plot of DEGs. (**C**) KEGG functional enrichment bar chart of DEGs. (**D**) Mapman annotation of DEGs.

In order to analyze the effect of CO_2_ enrichment on the expression of growth and development-related genes in cucumber leaves, transcriptome sequencing was performed on cucumber leaves under different CO_2_ conditions. By analyzing the DEGs annotated using transcriptome sequencing, a total of 1582 DEGs were identified in the leaves that were enriched with CO_2_ and the control, of which 1064 DEGs were upregulated and 518 DEGs were downregulated (Figure 3A, Table S1). Transcriptome sequencing was performed on DEGs using GO functional annotation and enrichment analysis. Firstly, it was found that chlorophyll biosynthesis and photosynthesis functions were significantly enriched, and most genes were significantly upregulated, indicating that CO_2_ enrichment promoted photosynthesis in cucumber leaves. Subsequently, it was found that carbohydrate metabolism, starch biosynthesis, maltose metabolism, phytosteroid metabolism, gluconic acid biosynthesis, and fatty acid metabolism pathways were significantly enriched. At the same time, functions related to leaf development were also significantly enriched, including leaf morphology development, indoleacetic acid biosynthetic, and hormone response (Figure 3B). The results indicated that CO_2_ enrichment may enhance photosynthesis to complete leaf tissue morphogenesis and growth through a series of carbohydrate metabolism, fatty acid metabolism, and auxin metabolism.

Through the KEGG metabolic pathway-enrichment analysis of DEGs, it was found that most genes were significantly enriched in photosynthesis, carbon metabolism, starch, and sucrose metabolism pathways. Specifically, there were three enriched pathways directly related to photosynthesis, and the number of enriched DEGs is 27, accounting for about 7.42%; the number of DEGs on the carbon metabolic pathway was 25, accounting for about 6.87%; and the number of genes on the starch and sucrose metabolic pathway was 22, accounting for about 6.04% (Figure 3C). A brief annotation analysis was conducted using Mapman on the entire metabolic pathway of DEGs, and it was found that the photosynthesis, carbohydrate metabolism, cell wall development, and lipid pathways were all annotated (Figure 3D). Among them, the photosynthesis pathway was annotated with the most DEGs, followed by the DEGs for light response, tetrapyrrole metabolism, and glutathione metabolism; glycolysis and the TCA were also annotated. CO_2_ enrichment has a significant impact on the photosynthesis, carbon fixation, carbohydrate metabolism, and fatty acid metabolism pathways of cucumber leaves, forming a complete metabolic regulation system for plant nutrient source-sink dynamics.

### 2.4. Analysis of Genes-Encoding Carbohydrate Metabolism Enzymes in Leaves under CO_2_ Enrichment

**Figure 4:**
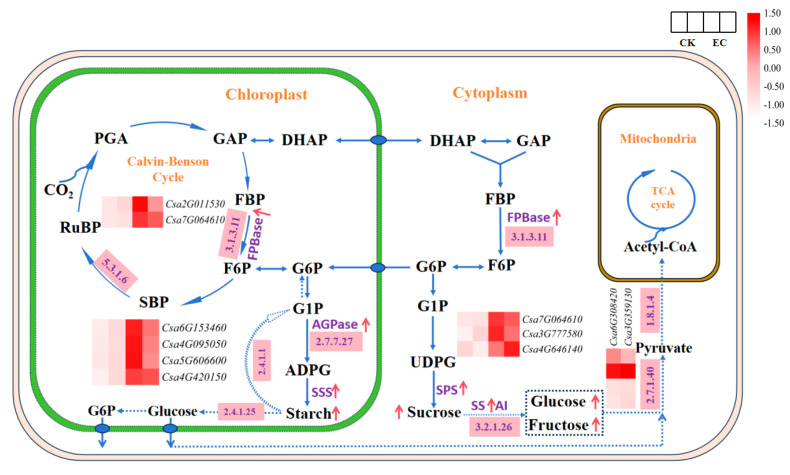
Enzymes activities and expression heatmap of genes related to the carbon metabolism pathway in cucumber leaves under CO_2_ enrichment. Solid blue arrows: carbohydrate synthesis pathway or substance transport; dashed blue arrows: carbohydrate catabolism pathway; pink boxes: key enzymes in metabolic pathways; red arrows: enzyme activity increased; and heatmap: expression of genes coding the key enzymes (FC ≥ 1.5). PGA, 3-phosphoglycerate; GAP, glyceraldehyde 3-phosphate; FBP, glyceraldehyde 3-phosphate; F6P, fructose 6-phosphate; SBP, glyceraldehyde 3-phosphate; RuBP, Ribulose-1,5-bisphosphate; G6P, glucose 6-phosphate; G1P, Glucose 1-phosphate; ADPG, Adenosine 5′-Diphosphoglucose; DHAP, dihydroxyacetone phosphate; GAP, glyceraldehyde 3-phosphate; UDPG, uridine diphosphate glucose.

Subsequently, genes relating to carbohydrate metabolism were screened, and a total of 23 key DEGs were identified in this study. These 23 key DEGs were significantly upregulated in CO_2_-enriched cucumber leaves, participating in carbon fixation, starch and sucrose metabolism, glycolysis, and tricarboxylic acid cycle (TAC) pathways (Table 1 and Figure 4). During the Calvin cycle, ribose 5-phosphate isomerase (Rpi) catalyzes the formation of ribulose-5-phosphate (Ru5P) from ribose 5-phosphate (R5P). The expression of *Csa2G011530* (Rpi) was 396.89 under CO_2_ enrichment, which was significantly higher than that of the control, 224.91, promoting the formation of RuBP and the carboxylation reaction. The expression of *Csa7G064610* (FBPase) was upregulated by 1.6-fold, promoting the formation of F6P. The results indicated that the high expression of two enzymes could promote the regulation of the Calvin cycle.

In the starch and sucrose pathway, *Csa6G153460* (AGPase) showed an expression level of 104.42 under CO_2_ enrichment, which was significantly higher than the control, 64.82. The expressions of *Csa5G606600*, *Csa4G095050* (starch phosphorylase), and *Csa4G646140*, *Csa3G777580* (β-fructosyltransferase) were upregulated 1.7–1.9-fold under CO_2_ enrichment, which indicated that CO_2_ enrichment promoted starch metabolism and sucrose decomposition. In the respiratory metabolism process, it was found that the expressions of *Csa3G359130* (pyruvate kinase), *Csa6G308420* (dihydrolipoamide dehydrogenasede), and *Csa2G373430* (malate dehydrogenase) under CO_2_ enrichment were significantly different from that of the control. Therein, *Csa6G308420* was upregulated by 2.1-fold after CO_2_ enrichment, indicating that high expression of the three genes can promote glucose glycolysis and TCA metabolism in leaf cells.

In addition, compared with the control, the expression of 10 genes encoding β-glucosidase, β-galactosidase, and pectinesterase also differed significantly under CO_2_ enrichment. Among them, the expression of *Csa2G337760* (β-galactosidase) was about 4-fold higher than that of the control. The expression of *Csa7G343850* (pectinesterase) was upregulated 2.8-fold compared with the control. The expression of *Csa1G042700* (β-glucosidase) after CO_2_ enrichment was 74.45, which was significantly higher than the control 44.6. These genes in response to CO_2_ enrichment were obvious, which may be important for the regulation of carbohydrate metabolism in cucumber leaves.

### 2.5. Correlation Analysis of Transcriptome and Metabolome

**Figure 5:**
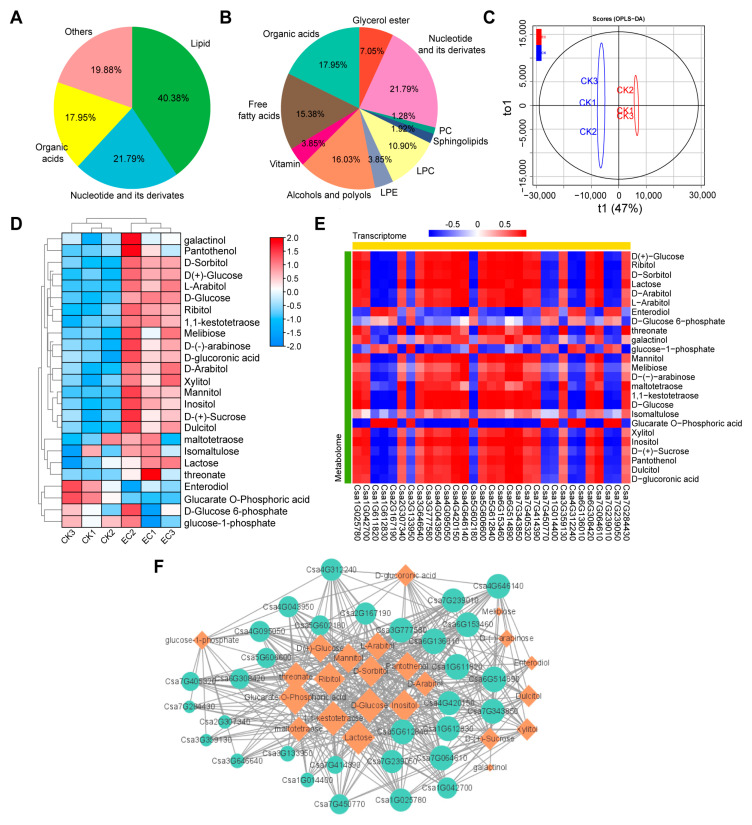
Identification of DAMs and key DEGs related to carbohydrate metabolism in cucumber leaves under CO_2_ enrichment. (**A**) Classification and abundance of primary compounds identified by the metabolome. (**B**) Classification and abundance of secondary compounds identified by the metabolome. (**C**) Identification of DAMs using OPLS-DA. (**D**) Heat map of the expression-related carbohydrate metabolism molecules. (**E**) Correlation heatmap of carbohydrate-related DEGs and DAMs. (**F**) Correlation network diagram of carbohydrate-related DEGs and DAMs. Orange diamonds show DAMs, blue circles show DEGs, gray lines show association.

In order to analyze the characteristics of metabolite changes in cucumber leaves under CO_2_ enrichment, metabolome measurements were subsequently performed on cucumber leaves. Through database functional annotation of the metabolites, it was found that the annotated metabolites mainly consisted of primary and secondary compound classifications. In the overall type of analysis, three classes of compounds were identified for primary compounds, for which lipid-related metabolic small molecules accounted for the highest proportion, about 40.38% of the total number of compounds, and nucleotides and their derivatives accounted for about 21.79% (Figure 5A). The identified secondary compounds were classified into 10 categories in detail, with sugars and alcohols accounting for a relatively high proportion in the secondary classification, about 16.03% of the total, and free fatty acids accounting for about 15.38% (Figure 5B). A total of 22 DAMs were identified using OPLS-DA, with 8 metabolites upregulated and 14 metabolites downregulated under CO_2_ enrichment (Figure 5C). Three carbohydrate and alcohol metabolites were identified via secondary annotation classification of DAMs (Table S2). D-anhydrous glucose and D(+)-anhydrous glucose significantly accumulated in cucumber leaves under CO_2_ enrichment, showing upregulation by 2.4-fold and 2.6-fold, respectively. Phosphogluconic acid was downregulated by 2.1-fold under CO_2_ enrichment (Figure 5D). By analyzing the content of DAMs, it was found that carbohydrate metabolites were mainly concentrated in the enriched carbon condition, indicating that CO_2_ enrichment promoted sugar accumulation in cucumber leaves.

Subsequently, the correlation analysis of DAMs and DEGs relating to carbohydrate metabolism was conducted, and at least 20 DEGs were found to have a highly positive correlation with the expression of D-glucose (Figure 5E). Among them, seven DEGs were found to be highly related to D-glucose, with a correlation of more than 0.93. The seven DEGs are as follows: *Csa7G064610* (FBPase), *Csa6G153460* (AGPase), *Csa4G420150* (4-α-glucantransferase), *Csa4G646140* (β-furanfrucoside), *Csa5G612840* (β-glucosidase), *Csa6G514890*, and *Csa7G343850* (pectinesterase). At the same time, by building a transcription-metabolic regulation network relating to sugar (Figure 5F), it was found that D-glucose correlation was the highest, and *Csa6G153460* (AGPase), *Csa5G612840* (β-glucosidase), and *Csa4G420150* (4-α-glucantransferase) are highly correlated under the regulatory network, which may play an important role in regulating carbohydrate metabolism in cucumber leaves under CO_2_ enrichment.

### 2.6. RT-qPCR Verification

**Figure 6:**
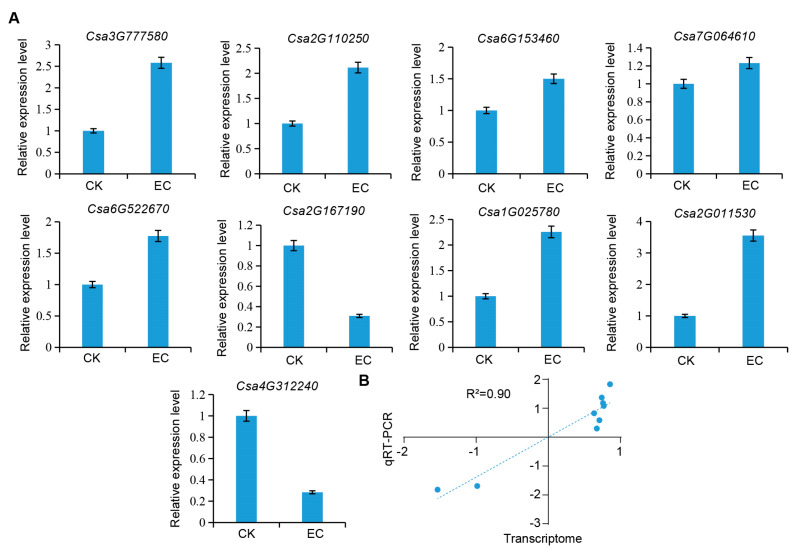
RT-qPCR validation of DGE results based on gene-expression levels. (**A**) RT-qPCR determination of candidate genes. (**B**) Correlation analysis between transcriptome and qPCR.

Nine DEGs relating to carbohydrate metabolism, *Csa3G777580*, *Csa2G110250*, *Csa6G153460*, *Csa7G064610*, *Csa6G522670*, *Csa2G167190*, *Csa1G025780*, *Csa2G011530*, and *Csa4G312240*, were screened and subjected to qPCR to analyze cucumber leaves under the CO_2_-enriched and control conditions. It was found that the expression trends of the nine genes were consistent with the sequencing results (Figure 6A). By conducting a correlation analysis of the transcriptome and qPCR results, a correlation coefficient of 0.9 was found, indicating the reliability of the transcriptome sequencing results (Figure 6B).

## 3. Discussion

CO_2_ enrichment can promote starch and sucrose metabolism. CO_2_ serves as a raw material for photosynthesis, and, to a certain extent, the amount of carbohydrate accumulation can directly reflect the intensity of plant photosynthesis, which is the degree of utilization of CO_2_. A study revealed that by artificially manufacturing chloroplasts to improve carbon fixation models, efficient conversion of CO_2_ into carbohydrates was achieved [24]. The content of soluble sugar and starch in Ningxia wolfberry fruit increased after 90 d of CO_2_ enrichment, and it is believed that the increased intercellular CO_2_ concentration helps to enhance the conversion and utilization of light energy in chloroplasts, thereby increasing the biomass of roots, stems, and fruits [25]. In this study, it was found that the number and volume of starch grains in chloroplasts increased, and starch and sucrose metabolism were significantly enriched in the GO function and KEGG pathway. It was identified that the expression level of *Csa6G153460* (AGPase) was significantly increased. Based on the results, it was evident that enriched CO_2_ activated AGPase expression and promoted starch accumulation in chloroplasts. However, some studies suggest that elevated CO_2_ leads to the accumulation of carbohydrates and downregulation of AGPase protein content in wheat leaves [26], which is not entirely consistent with this study. It was suggested that different species have different responses to elevated CO_2_ and may be related to regulating the balance between source and sink, which requires further study. Starch phosphorylase (SP) is a reversible enzyme that regulates chloroplast homeostasis. SP is a key intermediate enzyme that regulates chloroplast homeostasis; its synthesis direction can extend the non-reducing ends of the a-1,4 glucose chain of starch, while its phosphorylation direction can catalyze the phosphorylation of α-1,4-glycosidic bonds in starch to produce glucose-1-phosphate (G1P), which is responsible for the phosphorylation of the glucose chain. Of course, the direction of the reaction is affected by substrate concentration [27]. A starch phosphorylase-deficient mutant *chl3* was identified in corn, and it was found that excessive starch accumulation in chloroplasts led to the yellowing of leaves [28]. In this study, *Csa5G606600* and *Csa4G095050* (SP) were identified as significantly different from the control under CO_2_ enrichment. It was preliminarily analyzed that CO_2_ enrichment could improve photosynthetic efficiency, leading to a significant accumulation of starch in mesophyll cells, activating the expression of starch phosphorylase, and promoting starch metabolism and transfer. According to reports, the Artificial Starch Assimilation Pathway (ASAP) has been established, which synthesizes starch using CO_2_ and hydrogen in a cell-free system. The conversion rate of starch is 8.5 times higher than that of corn [29]. Further study of the function of genes involved in starch metabolism may provide new insights into ASAP. In addition, for sucrose metabolism, the activities of two enzymes, SPS and SS, were significantly increased, while their genes were not found to be significantly expressed after CO_2_ enrichment. It was also found that the expressions of *Csa4G646140* and *Csa3G777580* (β-fructofuranosidase) were significantly different from the control under CO_2_ enrichment. It was inferred that high expression of the invertase genes improved fructose transfer activity. Thus, CO_2_ enrichment promoted the decomposition of sucrose into fructose and glucose and avoided the overaccumulation of sucrose. It was inferred that the development of sink organs requires more material and energy, which was beneficial for regulating the relationship between source and sink. As a result, we can further investigate the function of the β-fructofuranosidase (Inv) gene, which will be beneficial to the research of leafy vegetable breeding.

CO_2_ enrichment has an effect on the carbon metabolism process. The core of photosynthetic carbon metabolism is the Calvin cycle, which includes intermediates of the Calvin cycle, starch synthesis precursor AGPase, phosphoenolpyruvate PEP, and pyruvate, forming a large metabolic activated carbon pool [30,31]. FBPase is one of the key enzymes regulating the Calvin cycle, with two types: cytoplasmic and chloroplastic. FBPase plays an important role in photosynthetic carbon assimilation and carbon allocation [32]. Overexpression of *Arabidopsis* FBPase in transgenic tobacco enhanced the regeneration of Ribulose 1,5-bisphosphate (RuBP), promoted CO_2_ fixation, and improved the growth and biomass in plants [33]. The expression level of chloroplast FBPase in *Arabidopsis* was reduced using antisense RNA technology while increasing the content of sucrose [34]. Overexpression of cytoplasmic FBPase in *Arabidopsis* significantly increased the rate of photosynthetic carbon assimilation and the content of soluble sugar [35]. This finding is consistent with the results of this study, as the expression level of FBPase was significantly upregulated under CO_2_ enrichment, and the starch content was significantly higher than that of sucrose. It is inferred that the expression level of chloroplast FBPase was significantly higher than that of cytoplasmic FBPase, and more carbon sources were allocated to starch. However, further research was required to investigate the differences between chloroplast FBPase and cytoplasmic FBPase in response to CO_2_. Pyruvate is the final product of glycolysis, and pyruvate kinase catalyzes the conversion of PEP to pyruvate. The pyruvate dehydrogenase system connects glycolysis and the TCA, controlling the pathway from pyruvate to acetyl CoA [36,37], and dihydrolipoamide dehydrogenase is a component of the pyruvate dehydrogenase system, which is involved in regulating the TCA in plants [38]. A study found that elevated CO_2_ could accelerate the accumulation of carbohydrates, phosphoglycerate (PGA), and phosphoenolpyruvate (PEP) in *Arabidopsis*, thereby enhancing respiratory potential [5]. In this study, it was found that glucose was significantly enriched in elevated CO_2_, and the expressions of pyruvate kinase and dihydrolipoamide dehydrogenase under CO_2_ enrichment were significantly different from the control. Moreover, the result indicated that elevated CO_2_ could catalyze the conversion of PEP to pyruvate via pyruvate kinase, accelerate the formation of acetyl CoA, promote the TCA, and enhance the enriched glucose to provide the energy required for the growth and development of cucumber leaves.

CO_2_ enrichment can affect cell wall development, and β-galactosidase, β-glucosidase, and β-fructofuranidase are associated with the development of cell walls. A study found that t *DkGAL1* (β-galactosidase) promoted the development of the pericarp cell wall in tomato [39]. Moreover, *BGAL1* and *BGAL3* (β-galactosidase) played a coordinated role in cell wall elongation in *Arabidopsis* [40]. In this study, it was found that the expression of *Csa2G337760* (β-galactosidase) was significantly upregulated under CO_2_ enrichment. Moreover, it was preliminarily found that CO_2_ enrichment promoted the release of more free galactose from galactoside-containing cell wall polysaccharides and remodeled cell walls. According to a study, *Os4BGlu18* (β-glucosidase) in rice could catalyze the conversion of monoinositol glycosides into monoinositol and aggregte into lignin to enhance plant cell walls [41]. It was found that the activity of β-glucosidase increased during the process of cell wall development in apricot fruit [42]. Moreover, *Os3BGlu6* (β-glucosidase) mutant in rice resulted in dwarf growth and lower photosynthetic capacity [43]. In this study, four β-glucosidase genes were identified with a significantly higher expression under CO_2_ enrichment, and it was inferred that the activation of β-glucosidase could promote cellulose metabolism and the formation of leaf cell walls, which is beneficial to leaf photosynthesis. In addition, pectinesterase promotes pectic acid formation and influences changes in cell wall polysaccharide composition. According to previous studies, watermelon *Cla004251* (pectinesterase) may be involved in the fortification and solubilization of pectin during fruit development, relating to changes in fruit firmness [44]. The expressions of *Ach15g378611* and *Ach14g026891* (pectinesterase) were inhibited in kiwi fruit under low-temperature ozone treatment, which significantly delayed the reduction in fruit firmness [45]. In this study, four pectinesterase genes were identified to be significantly upregulated under CO_2_ enrichment, which inferred that CO_2_ enrichment strengthened intercellular adhesion and favored cell wall component changes and metabolism. At the same time, this result coincided with the increase in leaf area and the number of mesophyll cells under CO_2_ enrichment, and the significant enrichment of GO functions, such as leaf tissue morphogenesis and cell wall development found in this study. It was further shown that CO_2_ enrichment led to the active expression of genes relating to cell wall development, and promoted the development and growth of mesophyll cells in cucumber.

## 4. Materials and Methods

### 4.1. Plants and Growth Conditions

The cucumber variety used was “Xinghai No. 7”, and the seeds were purchased from Xinghai Agricultural Co., Ltd. (Jiexiu, Shanxi, China). The experiment was conducted from January to May 2017 in the greenhouse of the Horticultural Station of Shanxi Agricultural University. An enriched CO_2_ zone EC (CO_2_ concentration of 800 ± 50 μmol·mol^−1^, denoted as elevated CO_2_) and a control zone CK (natural environment of 400 ± 50 μmol·mol^−1^, denoted as ambient CO_2_) were set up in the greenhouse, and separated using plastic film. A CO_2_ automatic release system was installed in the carbon-enriched zone with a liquid CO_2_ cylinder as the gas source. CO_2_ release was monitored using a GMM220 sensor (VAISALA company, Finland) and an automatic control system (Shengyan Electronic Science and Technology Co., Ltd., Handan, China). Cucumber seedlings with three true leaves were planted on 15 January, with a spacing of about 40 cm × 50 cm and 15 plants per row, 90 plants per treatment. Half a month after planting, the cucumber seeding received CO_2_ enrichment from 8:30 a.m. to 10:30 a.m. on sunny days, but not on snowy or rainy days, and the CO_2_ application time was a total of 50 d. The rest was routine cultivation management. The temperature was 15–32 °C; the humidity was 75–85%; watering was once every 7–10 days, or 5–7 days; twice water, once fertilizer, fertilizing followed with the watering.

### 4.2. Material Selection and Sampling

Before sampling, cucumber plants with uniform growth, no disease, and intact leaves were selected from the control zone and the carbon-rich zone, respectively. On the 14th day of CO_2_ enrichment, the first spread leaf of uniform size below the growth point of five plants was marked as the observation object for leaf growth dynamics observation. On the 30th day of CO_2_ enrichment, the leaves were marked as test subjects at the 2nd, 4th, 6th, 8th, and 10th nodes below the growing points of the three plants. A small square measuring 5 mm × 5 mm was quickly cut on both sides of the main vein of the test subject leaves and immediately put into the prepared FAA fixing solution for microscopic observation. Meanwhile, the same leaves were cut at the 4th node and the samples were immediately put into a 3% glutaraldehyde fixing solution, which was pumped until the samples were submerged and used for ultramicroscopic observations. All the material samples were brought back to the laboratory.

On the 21st, 35th, and 49th days of CO_2_ enrichment, the fourth functional leaf below the growing point on 18 plants was labeled as a test subject. Each sample was taken from three different plants and repeated three times. The intact fourth functional leaves were removed from nine plants and brought back to the laboratory for drying to determine soluble sugar and starch content. For the other nine plants, the mesophyll tissue was quickly cut on both sides near the main vein and wrapped with tin foil. The samples were immediately frozen in liquid nitrogen and stored at −80 °C for the determination of enzyme activity related to carbohydrate metabolism. Among them, CO_2_ enrichment samples obtained on the 35th day were used for the determination of monosaccharide and disaccharide contents, and RNA-seq, metabolome, and quantitative real-time PCR experiments were conducted.

### 4.3. Observation of Leaf Growth Dynamics

The leaf length (a) and leaf width (b) measurements of leaves were obtained using a ruler every 3 days until the 35th day after CO_2_ enrichment, recording the growth of the marked leaves. The leaf length was measured from the base to the tip, and the leaf width was measured from the widest part of the leaf. The leaf area was calculated and, finally, the average value was determined. The leaf area calculation formula was as follows [46]:

S = 14.61 − 5.0 (a) ± 0.94 (a^2^) ± 0.47 (b) ± 0.63 (b^2^) − 0.62 (a × b)

a indicates leaf length; b indicates leaf width.

### 4.4. Microscopic and Ultramicroscopic Observations

The material fixed in FAA fixative for 24 h was removed and then paraffin slice production was conducted with reference to the method reported by the authors [47]. The sections were observed under an OLYMPUS BX50 microscope (Olympus Corporation, Tokyo, Japan) and photographed. Next, a ruler was added with the image analysis software Image-pro 6.3 to measure the length and width of the fenestrated cells. The material fixed in glutaraldehyde for 2 days was removed and rinsed three times with phosphate buffer (pH 7.2) for electron microscopy slice preparation with reference to Shi and Liu’s methods, respectively [48,49], Then, these slices were observed and photographed under a scanning electron microscope JSM-6490LV (JEOL Ltd., Tokyo, Japan) and a transmission electron microscope JEM-1400 (JEOL Ltd., Tokyo, Japan), respectively.

### 4.5. Determination of Soluble Sugar and Starch Content

An amount of 0.1 g of each sample was combined with 80% ethanol solution. The material was ground and then placed in an 80 °C water bath for 30 min, with extraction of the supernatant conducted twice. Then, the supernatant was transferred to a 10 mL centrifuge tube and centrifuged for 10 min at 5000 rpm and 4 °C. The supernatant was the extracted soluble sugar solution, and the residue was used to determine the starch. The contents of soluble sugars and starch in the leaves were determined using anthrone colorimetry, using a Visible Spectrophotometer 723 (Shanghai Spectrum Instruments Co., Ltd., Shanghai, China) with reference to Zhang’s method [50]. The calculation formula was as follows:

Soluble total sugar content = [(C × Vt × n)/(W × Vs × 1000)] × 100%

Starch content = [(C × Vt × 0.9)/(W × Vs × 1000)] × 100%

where C is the glucose content (μg) as checked or calculated from the standard curve, n is the dilution multiple, 1000 is the conversion factor, Vt is the total volume of the sample extraction (mL), Vs is the sampling volume at the time of measurement (mL), W is the sample weight (mg), and 0.9 is the factor of glucose converting to starch.

### 4.6. Determination of Sucrose, Glucose, and Fructose Cotent

An amount of 0.5 g of each fresh sample was fully ground and placed into a 10 mL centrifuge tube, which was then extracted with ultrapure water for 30 min and centrifuged at 4000 rpm. The filtrate was fixed to 25 mL volumetric flask, and then 7–8 mL was filtered through an aqueous microporous membrane into sample bottles. The composition and content of monosaccharides and disaccharides were determined using an ion chromatograph ICS-3000 (Dionex Corporation, Sunnyvale, CA, USA) [51].

### 4.7. Determination of Enzyme Activities

An amount of 0.1 g of each fresh sample was fully ground with phosphate buffer (PH 7.4), placed into a 10 mL centrifuge tube, and then centrifuged at 4000 r·min^−1^. The supernatant was placed into a 1.5 mL centrifuge tube as a spare. The enzyme-linked immunosorbent assay (ELISA) was conducted and the absorbance (OD value) was measured at a wavelength of 450 nm using a Rayto RT-6100 analyzer (Rayto Life and Analytical Sciences Co., Ltd., Shenzhen, China). The enzyme activity in the sample was calculated using a standard curve [52].

### 4.8. RNA Extraction and Transcriptome Analysis

The extraction and RNA-Seq of total RNA were performed by Biomarker Technologies Co, Ltd. (Beijing, China), and the sequencing steps, expression analysis, and functional annotation methods were the same as those of Sun [53], based on cucumber (Chinese Long) genome v2 (http://cucurbitgenomics.org/, accessed on 10 September 2022). DGE screening was performed using DESeq2 (3.19) software (https://bioconductor.org/packages/release/bioc/html/DESeq2.html, accessed on 10 September 2022), and the screening criteria for DGEs were as follows: |FC| ≥ 1.5, FDR ≤ 0.05 [54]. GO functional enrichment analysis and KEGG metabolic pathway-enrichment analysis of DEGs were performed using TBtools_(v1_09867) software (https://github.com/CJ-Chen/TBtools-II/releases, accessed on 10 September 2022), and the Mapman (3.5.1) software (https://mapman.gabipd.org/, accessed on 10 September 2022) was used for functional annotation and visualization of the DEGs [55].

### 4.9. Determination and Analysis of Metabolome

After vacuum freeze-drying cucumber leaves, they were ground to powder using a grinding mill MM400 (Retsch, German) at 30 Hz. An amount of 100 mg was dissolved in 0.6 mL of extraction solution and left overnight at 4 °C in a refrigerator. During this period, the cucumber leaves were vortexed six times in batches to improve extraction efficiency. Subsequently, the leaves were centrifuged at 10,000 rpm for 10 min, and the supernatant was extracted. The sample was filtered through a microporous membrane (0.22 μm pore size) and stored in an injection bottle for machine-sequencing analysis. Metabolite identification and analysis were performed by Metware Biotechnology Co., Ltd. (Wuhan, China). DAM identification was performed using OPLS-DA, in which a combination of |log_2_FC| ≥ 1, *p*-value ≤ 0.05, and VIP ≥ 1 was adopted to screen for DAMs. Then, the Spearman correlation was used to analyze the correlation between DAMs and DEGs [56].

### 4.10. Quantitative Real-Time PCR

To validate the RNA-sequencing results, quantitative real-time PCR (qRT-PCR) was performed using gene-specific primers for nine randomly selected genes. Primer 3 software was used to design specific primers. The Tubulin Alpha Chain (TUA) cucumber gene was used as a reference gene. Total RNA from the fourth leaf of the control and the CO_2_-enriched sample was extracted, using an RNeasy Plant Mini Kit (QIAGEN, 74903, Dusseldorf, North Rhine-Westphalia, Germany). First-strand cDNA was obtained using a PrimeScript^®^ RT reagent kit (Perfect Real Time, TaKaR a, RR037A, Tokyo, Japan). qRT-PCR was conducted using SYBR^®^Premix Ex Taq™ II (Tli RNaseH Plus, TaKaRa, Tokyo, Japan) with an ABI 7500 instrument (Applied Biological Inc., Massachusetts, Waltham, USA). The 25 μL reaction included 20 ng of cDNA. The program was as follows: 94 °C for 1 min; 40 cycles of 94 °C for 30 s, 55 °C for 30 s, and 72 °C for 30 s. The relative expression levels were calculated using the 2^−ΔΔCT^ method [57]. The primer sequences used for the genes are shown in Table S3.

### 4.11. Statistical Analysis

Statistical analyses were conducted using IBM SPSS Statistics 25 software. Asterisks indicate significant differences using the two-tailed Student’s *t*-test (* *p*  <  0.05, *** *p  *<  0.001, and **** *p*  <  0.0001). Significant differences indicated by different letters were calculated using Duncan’s new multiple-range test.

## 5. Conclusions

**Figure 7:**
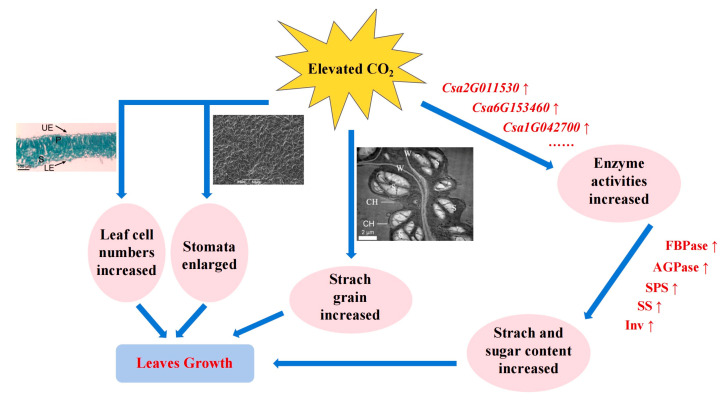
Mechanism diagram of CO_2_ promotion of leaf growth. Blue arrows show the direction of the results caused by the mechanism reaction, red arrows show increased facilitation.

In summary, CO_2_ enrichment enhanced the photosynthetic capacity, improved their tissue structure, and promoted enzyme activity and carbohydrate accumulation in the leaves (Figure 7). The photosynthesis, carbon metabolism, starch, and sucrose metabolism were found to be significantly enriched in the GO function and KEGG pathway. 23 DEGs related to carbon metabolism were involved in the regulation of major metabolism pathways, such as the Calvin cycle, starch and sucrose metabolism, glycolysis, and TAC. Moreover, DAMs were mainly concentrated in carbohydrate; *Csa7G064610* (FBPase), *Csa6G153460* (AGPase), *Csa4G420150* (4-α-glucanotransferase), *Csa4G646140* (Inv), *Csa5G612840* (β-glucosidase), *Csa6G514890,* and *Csa7G343850* (pectinesterase) were highly correlated with D-glucose. Furthermore, the mechanism of CO_2_ enrichment that promotes carbohydrate metabolism in leaves at the molecular level was revealed. This mechanism advances the development of the cell wall and leaf morphology by activating the expression of key genes and improving enzyme activity.

## Figures and Tables

**Table 1:** Expression of key enzyme genes in the process of carbon metabolism.

Enzyme ID	Enzyme Name	Gene ID	FPKM		Expression Pattern
5.3.1.6	ribose-5-phosphate isomerase	*Csa2G011530*	224.92	396.89	Up
3.1.3.11	fructose-1,6-bisphosphatase	*Csa7G064610*	13.94	21.90	Up
2.7.7.27	ADP-glucose pyrophosphorylase	*Csa6G153460*	64.82	104.42	Up
2.4.1.1	starch phosphorylase	*Csa4G095050*	14.42	26.24	Up
		*Csa5G606600*	10.84	20.61	
3.2.1.21	β-glucosidase	*Csa1G025780*	10.33	17.38	Up
		*Csa5G612840*	2.13	4.25	
		*Csa1G042700*	44.60	74.45	
3.2.1.26	β-fructofuranosidase	*Csa3G777580*	8.56	14.14	Up
		*Csa4G646140*	5.53	8.96	
3.2.1.23	β-galactosidase	*Csa3G865330*	6.51	12.97	Up
		*Csa6G504610*	17.43	28.51	
		*Csa2G337760*	3.97	16.10	
2.4.1.43	α-1,4-galacturonosyltransferase	*Csa6G075210*	17.48	26.45	Up
		*Csa1G179740*	17.16	25.49	
3.1.1.11	pectinesterase	*Csa7G343850*	1.41	3.89	Up
		*Csa6G514890*	2.70	5.19	
		*Csa3G646640*	32.93	60.06	
		*Csa7G414390*	23.30	39.99	
2.4.1.25	4-α-glucanotransferase	*Csa4G420150*	9.29	16.03	Up
2.7.1.40	pyruvate kinase	*Csa3G359130*	19.47	30.87	Up
1.8.1.4	dihydrolipoamide dehydrogenase	*Csa6G308420*	25.75	62.13	Up
1.1.1.40	malate dehydrogenase	*Csa2G373430*	19.66	34.77	Up

## Data Availability

Data are contained within the article and Supplementary Materials. Further information or data can be available from the corresponding author upon request.

## Supplementary Materials

The following supporting information can be downloaded at: https://www.mdpi.com/article/10.3390/ijms252011309/s1.
